# Sexual consent and chemsex: a quantitative study on sexualised drug use and non-consensual sex among men who have sex with men in Amsterdam, the Netherlands

**DOI:** 10.1136/sextrans-2020-054840

**Published:** 2021-04-08

**Authors:** Susanne Drückler, Jilke Speulman, Martijn van Rooijen, Henry J C De Vries

**Affiliations:** 1 Infectious Diseases, Public Health Service of Amsterdam, Amsterdam, The Netherlands; 2 Department of Dermatology, Amsterdam University Medical Centres, Amsterdam, The Netherlands

**Keywords:** sexual health, sexual behavior, risk factors, public health, health promotion

## Abstract

**Background:**

Chemsex (drug use to enhance sex) has emerged among men who have sex with men (MSM). Non-consensual sex (NCS) is hypothesised to occur frequently under the influence of chemsex, however data are scarce. In this cross-sectional study, it was aimed to assess whether NCS is associated with chemsex.

**Methods:**

We offered a survey about chemsex in the past 6 months (crystal methamphetamine, mephedrone and/or gamma-hydroxybutyrate/gamma-butyrolacton use) and NCS (sexual experiences beyond one’s limits or unpleasant sexual experiences) in the past 5 years to Amsterdam-located gay dating platform users. Associations were assessed using χ² test, Fisher’s exact test and multivariable logistic regression.

**Results:**

Of 891 participants, 30.6% (273 of 891) engaged in chemsex; 21.2% engaging and 16.7% not engaging in chemsex reported any NCS experiences (p=0.109).

Among MSM who reported any NCS experiences, chemsex engagers reported being touched against one’s will less often compared with non-engagers (22.4% vs 39.8%; p=0.036). Yet, chemsex engagers reported passing out and not remembering what happened during drug use more often (41.4% vs 8.7%; p<0.001). The level of suffering from NCS experiences did not differ between chemsex engagers and non-engagers (p=0.539); and was rated by most participants with no suffering at all or low suffering (77.1%). In the multivariable regression analyses, chemsex engagement in the past 6 months was associated with NCS (adjusted OR 1.46; 95% CI 1.01 to 2.11).

**Conclusions:**

A substantial proportion of MSM (regardless of chemsex engagement) reported NCS in the past 5 years. In multivariate logistic regression analysis, chemsex engagement was associated with an NCS experience. Among participants who reported NCS, suffering related to NCS however, did not differ between chemsex engagers and non-engagers. Sexual healthcare professionals need to address chemsex and NCS during consultations involving MSM and refer men for specialised help if deemed necessary.

## Introduction

Over the last decade, chemsex has become a rising phenomenon in the gay community.^1–3^ While there are different definitions of chemsex, here we define it as the use of any combination of the following drugs: crystal methamphetamine, mephedrone, and/or gamma-hydroxybutyrate/gamma-butyrolacton (GHB/GBL) before or during sex by men who have sex with men (MSM).^4^ Chemsex can also include different or more drugs depending on the social context or geographical region.^5^ The majority of MSM engaging in chemsex do not self-identify with problematic drug use and hardly report any disadvantages in everyday life.^6 7^ Yet, some report a negative impact on their life and pressure from friends to engage in chemsex.^8^ Moreover, chemsex engagement is associated with sexual risk-taking behaviour such as condomless anal sex, and may possibly lead to negative physical and mental health consequences and addiction.^1 9^ Amsterdam, among other big European cities, has shown a high prevalence of chemsex.^9 10^ Rising numbers in European countries like England, Belgium and the Netherlands are of concern, especially with regard to crystal methamphetamine use which is known to be highly addictive and can produce acute and chronic medical and psychiatric conditions.^11–13^

The meaning of chemsex engagement does not take into account the subculture and multifactorial issues surrounding the phenomenon of chemsex; for example, gay online dating culture and the fine line between consensual and non-consensual sex (NCS) are tightly linked to chemsex engagement and have become a more discussed topic in recent years.^14–16^ Chemsex engagement often takes place at home during sexualised parties with a small number of men who have met through dating applications or websites.^17–19^ A qualitative study reported that giving a notion of consent in a chemsex environment (characterised by sexually thrilling and loss of sexual desire control) felt difficult for some men.^14^

There are many variations in terms for NCS, such as sexual harassment, molestation and forced sex; here we use the term NCS to address all sexual experiences beyond one’s limits or unpleasant sexual experiences. In the Netherlands, approximately 3.8% of men, regardless of sexuality, experienced oral/anal penetration without consent at least once in their life.^20^ Moreover, the prevalence of NCS is higher among gay and bisexual men, compared with heterosexual men.^21 22^

Sexual consent is a human right, yet when drugs are involved it oftentimes becomes more difficult to consent and sexually transgressive behaviour seems to occur more easily.^23^ NCS among MSM—and especially during chemsex engagement—has been underexamined in research. Moreover, most research concerning chemsex has (sexual) health outcomes as main focus. Therefore, here we aimed to study chemsex engagement in relation to NCS. So we assessed whether NCS is associated with chemsex engagement.

## Method

### Data collection and study population

Users of two online gay dating platforms (Grindr and Gayromeo) were invited to participate in an anonymous online survey through interstitial advertisement. The survey was online on both platforms until the paid budget was depleted and was only shown to users in the Amsterdam area, based on their Global Positioning System location/Internet Protocol address. As we provided the survey sequentially on two different platforms–starting with Grindr first in August 2018 during Pride Amsterdam, Gayromeo users who answered that they previously filled in the survey were excluded from the analysis.

### Sociodemographic characteristics

Sociodemographic data we collected were: age, gender, sexuality (having sex with men, women, both) and residency (Amsterdam, elsewhere in the Netherlands, abroad). We furthermore asked whether one had been tested for STI/HIV in the past year and if they had tested at the Public Health Service of Amsterdam or elsewhere.

### Chemsex engagement characteristics

Chemsex engagement was defined as the use of any of the following drugs before or during sex in the past 6 months: crystal methamphetamine, mephedrone, and/or GHB/GBL. Chemsex-specific characteristics that were asked included the frequency of chemsex per substance (once per month or less, 2–4 per month, 2–3 per week, ≥4 per week), frequency of condomless insertive and/or receptive anal sex during chemsex, last time having sex without any of the chemsex-associated drugs (sober sex), intravenous drug use and if so, whether needles were shared.

### NCS characteristics

NCS was defined at the beginning of the survey as ‘sexual experience(s) where someone went beyond your limits or where you had an unpleasant experience’; this was explained with four examples: being filmed/photographed without permission, being touched against my will, had sex against my will, passed out and did not know what happened.

We asked participants whether they have had an NCS experience in the past 5 years. Then we instructed participants to think of the last NCS experience they had, we asked when those experiences happened and whether it was under the influence of chems.

To specify their own last NCS experience, participants were given the option of eight different types and one write-in option: (1) I have been filmed/photographed without permission, (2) I am blackmailed (eg, with images), (3) I have been given drugs (chems or other substances) against my will, (4) I have been touched against my will, (5) I have had sex against my will, (6) I had sex without a condom against my will, (7) I passed out (chems, other substances and/or alcohol) and do not remember what happened, (8) I have exceeded the limit of my sex partner.

Lastly, we asked for the current amount of emotional suffering due to one’s last NCS experience using a 5-point Likert scale in order to allow the participant to express how much they still emotionally suffer. Given the sensitive nature of the subject, we offered participants two different services where they could seek help regarding sexual assault.

### Statistical analysis

Using the χ² test for independence and Fisher’s exact test, we compared demographics and NCS experiences among participants who engaged in chemsex with those who did not. Using the χ² test for independence and the Fisher’s exact test, we compared demographics and chemsex behaviour between men having experienced NCS with those who did not. To investigate associations of chemsex engagement with NCS, a multivariable backward logistic regression analysis was done including all variables that were associated with NCS at p≤0.10 in the univariable analysis. Chemsex engagement was forced into the model as it was the variable of interest. Additionally, the multivariable model was repeated three times to test whether the three different types of drugs were independent variables for NCS. P<0.05 was considered statistically significant. Data were collected with LimeSurvey and data analyses were performed using IBM SPSS Statistics V.21.0.

## Results

**Figure 1:**
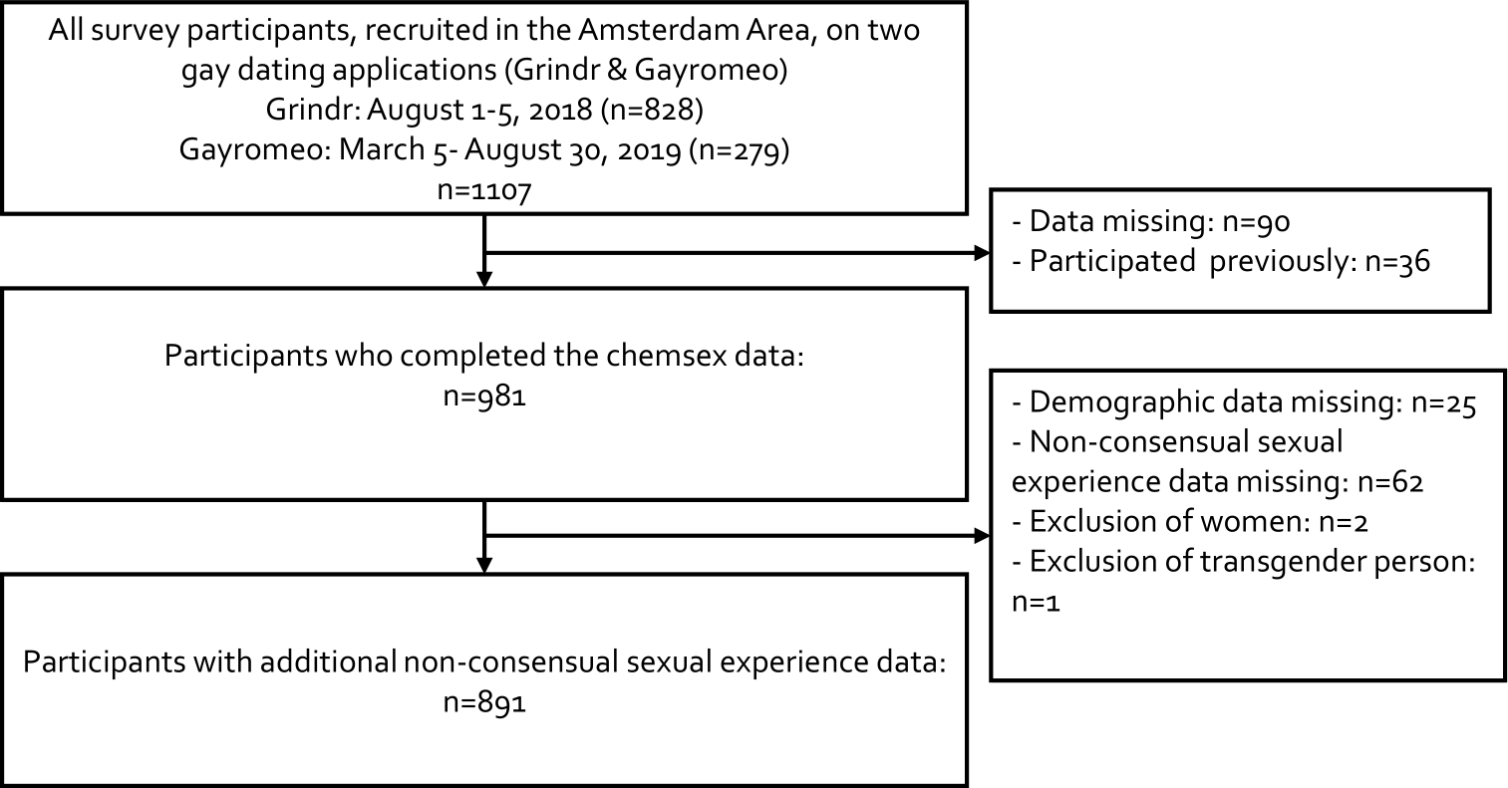
Flow chart of participants in an online survey on chemsex behaviour and non-consensual sexual experiences among men who have sex with men, Amsterdam, the Netherlands, 2018–2019.

Between August 2018 and August 2019, a total of 1107 (Grindr n=828 and Gayromeo n=279) participants started the survey. Due to missing data on chemsex and/or NCS experience, previously filled in surveys, the exclusion of women and one transgender person, n=216 participants were excluded and n=891 were included in the analyses (figure 1). The median age of participants was 39 years (IQR: 30–50), 46.3% (412 of 891) had their residency in Amsterdam and 93.2% (830 of 891) reported sex with men exclusively (table 1).

**Table 1:** Demographics and non-consensual sexual* experiences of 273 MSM who practised and 620 MSM who did not practise chemsex† in the past 6 months, Amsterdam area, the Netherlands, between August 2018 and August 2019

Variables	Chemsex† yes, n=273 (30.6%), n (%)	Chemsex† no, n=618 (69.4%), n (%)	P value	Total n=891 (%), n (%)
Median age in years (IQR)	40 (31–50)	39 (29–49)	0.071	39 (30–50)
Age categories in years			0.054	
≤24	22 (8.1)	76 (12.3)		98 (11.0)
25–34	72 (26.4)	174 (28.2)		246 (27.6)
35–44	66 (24.2)	138 (22.4)		204 (22.9)
45–54	75 (27.5)	155 (25.0)		230 (25.8)
55–64	36 (13.2)	57 (9.2)		93 (10.4)
≥65	2 (0.7)	18 (2.9)		20 (2.2)
Residency (missing n=1)			0.004	
Amsterdam	106 (38.8)	306 (49.6)		412 (46.3)
Elsewhere in the Netherlands	164 (60.1)	298 (48.3)		462 (51.9)
Abroad	3 (1.1)	13 (2.1)		16 (1.8)
Sex in <6 months with			0.706	
Men	253 (92.7)	577 (93.4)		830 (93.2)
Both men and women	20 (7.3)	41 (6.6)		61 (6.8)
STI/HIV test in the last year			<0.001	
No	21 (7.7)	170 (27.5)		191 (21.4)
Yes, at the Public Health Service of Amsterdam	71 (26.0)	183 (29.6)		254 (28.5)
Yes, elsewhere	181 (66.3)	265 (42.9)		446 (50.1)
Condomless anal chemsex	202/273 (74.0)	n.a.		
Frequency				
Always without	72 (35.6)	n.a.		
Most of the time without	53 (26.2)	n.a.		
Sometimes with	38 (18.8)	n.a.		
Most of the time with	39 (19.3)	n.a.		
Always with	0	n.a.		
Non-consensual sexual experience* in the past 5 years				
Yes	**58**/273 (21.2)	**103**/618 (16.7)	0.109	**161**/891 (18.1)
Timeframe (missing n=3)			0.002	
Past month	9/56 (16.1)	4/102 (3.9)		13/158 (8.2)
1–3 months ago	12/56 (21.4)	7/102 (6.9)		19/158 (12.0)
3–6 months ago	7/56 (12.5)	12/102 (11.8)		19/158 (12.0)
6–12 months ago	6/56 (10.7)	14/102 (13.7)		20/158 (12.7)
>1 year ago	22/56 (39.3)	65/102 (63.7)		87/158 (55.1)
	**Chemsex† yes and NCS yes, n=58 (36.0%),** **n (%**)	**Chemsex† no and NCS yes, n=103 (64.0%),** **n (%**)	**P value**	**Total n=161 (%),** **n (%**)
**Types of experience (multiple answers possible**)				
Filmed/photographed without consent	15 (25.9)	14 (13.6)	0.058	29 (18.0)
Blackmailed (eg, with images)	4 (6.9)	4 (3.9)	0.460	8 (5.0)
Used drugs against my will	10 (17.2)	9 (8.7)	0.130	19 (11.8)
Touched against my will	13 (22.4)	41 (39.8)	0.036	54 (33.5)
Had sexual contact against my will	17 (29.3)	37 (35.9)	0.487	54 (33.5)
Had sex without a condom against my will	12 (20.7)	26 (25.2)	0.566	38 (23.6)
I passed out and don’t remember (under influence of chems, other substances and/or alcohol)	24 (41.4)	9 (8.7)	<0.001	33 (20.5)
I crossed the limits of my sex partner	4 (6.9)	8 (7.8)	0.999	12 (7.5)
Other	1 (1.7)	5 (4.9)	0.420	6 (3.7)
**Current level of suffering** (missing n=4)			0.539	
- **-** no suffering	25 (44.6)	36 (35.6)		61 (38.9)
- **low suffering**	18 (32.1)	42 (41.6)		60 (38.2)
-+ **medium suffering**	8 (14.3)	14 (13.9)		22 (14.0)
**+ a bit suffering**	4 (7.1)	4 (4.0)		8 (5.1)
**++** suffering a lot	1 (1.8)	5 (5.0)		6 (3.8)

*Non-consensual sexual experience was defined as ‘where someone went beyond one’s limits or where one had an unpleasant experience’.

†The use of any combination of drugs, including crystal methamphetamine, mephedrone, and/or gamma-hydroxybutyrate/gamma-butyrolacton before or during sex in past 6 months.

MSM, men who have sex with men; n.a., not applicable; NCS, non-consensual sex.

Of all participants, 30.6% (273 of 891) engaged in chemsex in the past 6 months.

### NCS experiences

Of all participants, 161 of 891 (18.1%) reported at least one NCS experience in the past 5 years (table 1). Overall, the group of participants engaging in chemsex and the group of participants who did not engage in chemsex did not differ in prevalence of NCS in the past 5 years (58 of 237, 21.2% vs 103 of 618, 16.7%; p=0.109). However, among participants who reported NCS, two specified types of NCS differed significantly between chemsex and non-chemsex engagers: (1) MSM who engaged in chemsex reported ‘being touched against one’s will’ less often (13 of 58; 22.4% vs 41 of 103; 39.8%; p=0.036). (2) MSM who engaged in chemsex reported ‘having passed out and not remembering what happened during drug use’ (all possible drugs and/or alcohol) more often (24 of 58; 41.4% vs 9 of 103; 8.7%; p<0.001).

Participants who engaged in chemsex and had NCS reported more recent NCS experiences than participants who did not engage in chemsex and had NCS (p=0.002); meaning most participants (65 of 102; 63.7%) in the non-chemsex group reported that their most recent NCS experience was longer than 1 year ago (chemsex group: 22 of 56; 39.3%).

Most participants (77.1%) rated the level of current suffering due to one’s last NCS on a 5-point Likert scale with no suffering at all (61 of 161; 38.9%) or low suffering (60 of 161; 38.2%) and this was not significantly different between participants who engaged in chemsex and who did not (p=0.539). However, 1.8% (1 of 58) of participants engaged in chemsex and 5.0% (5 of 103) of participants did not rate their suffering with the highest possible option (suffering a lot). In online supplemental tables 1 and 2, we display the amount of suffering in relation to the different types of NCS and timeframe.

10.1136/sextrans-2020-054840.supp1 Supplementary data

### NCS experiences and chemsex characteristics

Participants who experienced NCS were younger (median 33 vs 40; p<0.001), more often crystal methamphetamine users (15.5% vs 8.5%; p=0.009) and reported more often intravenous drug use (6.2% vs 2.1%; p=0.008) compared with participants who did not experience NCS (table 2).

**Table 2:** Chemsex* behaviour and drug used, among 161 MSM who reported non-consensual sexual experience† and 730 MSM who did not, in Amsterdam, the Netherlands between August 2018 and August 2019

Variables	Non-consensual sexual experience†, yes n=161 (18.1%), n (%)	Non-consensual sexual experience†, no n=730 (81.9%), n (%)	P value	Total n=891 (%), n (%)
Median age (IQR), years	33 (25–46)	40 (30–50)	<0.001	39 (30–50)
Age categories, years (missing n=2)			<0.001	
≤24	36 (22.4)	62 (8.5)		98 (11.0)
25–34	49 (30.4)	197 (27.0)		246 (27.6)
35–44	29 (18.0)	175 (24.0)		204 (22.9)
45–54	34 (21.1)	196 (26.8)		230 (25.8)
55–64	11 (6.8)	82 (11.2)		93 (10.4)
≥65	2 (1.2)	18 (2.5)		20 (2.2)
Sex in <6 months with			0.003	
Men	141 (87.6)	689 (94.4)		830 (93.2)
Both men and women	20 (12.4)	41 (5.6)		61 (6.8)
Residency (missing n=1)			0.729	
Amsterdam	78 (48.4)	334 (45.8)		412 (46.3)
Outside of Amsterdam	81 (50.3)	381 (52.3)		462 (51.9)
Abroad	2 (1.2)	14 (1.9)		16 (1.8)
STI/HIV test in the last year			0.561	
No	30 (18.6)	161 (22.1)		191 (21.4)
Yes, at the Public Health Service of Amsterdam	50 (31.1)	204 (27.9)		254 (28.5)
Yes, somewhere else	81 (50.3)	365 (50.0)		446 (50.1)
Chemsex* <6 months	58 (36.0)	215 (29.5)	0.109	273 (30.6)
GHB/GBL (G) use during sex <6 months	53 (32.9)	200 (27.4)	0.176	253 (28.4)
Frequency			0.053	
Once per month or less	22/53 (41.5)	120/200 (60.0)		142/253 (56.1)
2–4 per month	23/53 (43.4)	67/200 (33.5)		90/253 (35.6)
2–3 a week	4/53 (7.5)	7/200 (3.5)		11/253 (4.3)
≥4 a week	4/53 (7.5)	6/200 (3.0)		10/253 (4.0)
Crystal methamphetamine (C) use during sex <6 months	25 (15.5)	62 (8.5)	0.009	87 (9.8)
Frequency			0.434	
Once per month or less	14/25 (56.0)	46/62 (74.2)		60/87 (69.0)
2–4 per month	8/25 (32.0)	12/62 (19.4)		20/87 (23.0)
2–3 a week	2/25 (8.0)	2/62 (3.2)		4/87 (4.6)
≥4 a week	1/25 (4.0)	2/62 (3.2)		3/87 (3.4)
Mephedrone (M) use during sex <6 months	20 (12.4)	47 (6.4)	0.013	67 (7.5)
Frequency			0.059	
Once per month or less	11/20 (55.0)	39/47 (83.0)		50/67 (74.6)
2–4 per month	5/20 (25.0)	4/47 (8.5)		9/67 (13.4)
2–3 a week	2/20 (10.0)	1/47 (2.1)		3/67 (4.5)
≥4 a week	2/20 (10.0)	3/47 (6.4)		5/67 (7.5)
Type of chemsex drugs used during sex <6 months‡			0.078	
G only	24/58 (41.4)	129/215 (60.0)		153/273 (56.0)
G and C	9/58 (15.5)	30/215 (14.0)		39/273 (14.3)
G and M	9/58 (15.5)	20/215 (9.3)		29/273 (10.6)
C and G and M	11/58 (19.0)	21/215 (9.8)		32/273 (11.7)
C only	5/58 (8.6)	9/215 (4.2)		14/273 (5.1)
M only	0/58 (0)	4/215 (1.9)		4/273 (1.5)
C and M	0/58 (0)	2/215 (0.9)		2/273 (0.7)
Injecting drugs <6 months	10 (6.2)	15 (2.1)	0.008	25 (2.8)
Shared needles	3/10 (30.0)	1/15 (6.7)	0.267	4/25 (16.0)
Sober sex last time§			0.045	
In the past month	36/58 (62.1)	166/215 (77.2)		202/273 (74.0)
Longer than 3 months ago	5/58 (8.6)	20/215 (9.3)		25/273 (9.2)
Longer than 6 months ago	5/58 (8.6)	11/215 (5.1)		16/273 (5.9)
Longer than 1 year ago	8/58 (13.8)	14/215 (6.5)		22/273 (8.1)
I do not remember	4/58 (6.9)	4/215 (1.9)		8/273 (2.9)
Condomless anal chemsex	44 (27.3)	161 (22.1)	0.178	205 (23.0

*The use of any combination of drugs, including crystal methamphetamine, mephedrone, and/or GHB/GBL before or during sex in past 6 months.

†Non-consensual sexual experience was defined as ‘where someone went beyond your limits or where you had an unpleasant experience’ (eg, filmed/photographed without consent, had sexual contact against their will, etc).

‡It is not known whether drugs were consumed at the same time or sequential.

§Last time having sex without any of the chemsex-associated drugs.

GHB/GBL, gamma-hydroxybutyrate/gamma-butyrolacton; MSM, men who have sex with men.

Participants who engaged in chemsex and reported NCS differed significantly from those who also engaged in chemsex but did not reported NCS as far as having had sober sex in the last month and not remembering the last time they had sober sex (overall p=0.045).

### Associations with NCS experiences

In the univariable analysis, chemsex engagement in the past 6 months was not associated with NCS in the past 5 years (OR 1.35; 95% CI 0.94 to 1.93) (table 3).

**Table 3:** Univariable and multivariable analyses of determinants associated with non-consensual sex* in the past 5 years among 891 MSM recruited on online dating apps between August 2018 and August 2019, the Netherlands

	Univariable, OR (95% CI)	P value	Multivariable, aOR (95% CI) Chemsex	P value	Multivariable, aOR (95% CI) Mephedrone	P value	Multivariable, aOR (95% CI) GHB/GBL	P value	Multivariable, aOR (95% CI) Crystal meth	P value
Age (IQR), years	0.96 (0.90 to 0.98)	<0.001	0.96 (0.95 to 0.98)	<0.001	0.97 (0.95 to 0.98)	<0.001	0.96 (0.95 to 0.98)	<0.001	0.96 (0.95 to 0.98)	<0.001
Condomless anal chemsex†		0.151	–	–	–	–	–	–	–	–
No	1									
Yes	1.33 (0.90 to 1.96)									
Sex in <6 months with		0.003		0.007		0.012		0.007		0.007
Men	1		1		1		1		1	
Men and women	2.38 (1.36 to 4.19)		2.21 (1.24 to 3.92)		2.10 (1.18 to 3.75)		2.20 (1.24 to 3.91)		2.22 (1.25 to 3.96)	
Chemsex† <6 months		0.102		0.044		–		–		
Not using chems during sex	1		1		–		–			
Using chems during sex	1.35 (0.94 to 1.93)		1.46 (1.01 to 2.11)							
Mephedrone (M) use <6 months		0.010	–	–		0.023	–	–		
Not using M during sex	1				1					
Using M during sex	2.06 (1.19 to 3.59)				1.93 (1.10 to 3.40)					
GHB/GBL (G) use <6 months		0.160	–	–	–	–		0.079		
Not using G during sex	1						1			
Using G during sex	1.30 (0.90 to 1.88)						1.40 (0.96 to 2.04)			
Crystal methamphetamine use (C) <6 months		0.007	–	–	–	–				0.003
Not using C during sex	1								1	
Using C during sex	1.98 (1.20 to 3.26)								2.18 (1.25 to 3.96)	

*Non-consensual sexual experience was defined as ‘where someone went beyond your limits or where you had an unpleasant experience’ (eg, filmed/photographed without consent, had sexual contact against their will).

†The use of any combination of drugs, including crystal methamphetamine, mephedrone, and/or GHB/GBL before or during sex.

aOR, adjusted OR; GHB/GBL, gamma-hydroxybutyrate/gamma-butyrolacton; MSM, men who have sex with men.

Whereas age (OR 0.96; 95% CI 0.90 to 0.98), having sex with both men and women, as opposed to with men only (OR 2.38; 95% CI 1.36 to 4.19), mephedrone use (OR 2.06; 95% CI 1.19 to 3.59) and crystal methamphetamine use in the past 6 months (OR 1.98; 95% CI 1.20 to 3.26) were significant determinants of NCS.

In the multivariable regression analyses, chemsex engagement in the past 6 months was associated with NCS (adjusted OR (aOR) 1.46; 95% CI 1.01 to 2.11), as well as younger age (aOR 0.96; 95% CI 0.95 to 0.98) and having sex with both men and women as opposed to men only (aOR 2.21; 95% CI 1.24 to 3.92). In the multivariable model for the three different types of drugs as independent variable for NCS, significant associations were found for mephedrone (aOR 1.93; 95% CI 1.10 to 3.40) and crystal methamphetamine use (aOR 2.18; 95% CI 1.25 to 3.96).

## Discussion

In this study, we found that a considerable proportion of MSM recently engaged in chemsex and had an NCS experience in the past 5 years. Moreover, in a multivariate logistic regression analysis, we established that chemsex engagement is associated with NCS. The advertisement strategy via online gay dating platforms gave insight in the Dutch chemsex subculture and the associations with NCS.

In our study, the overall prevalence of NCS was 18.1% in the past 5 years. A bit less than the half (44.9%) of all experiences was within the past year. In an earlier report on sexual violence (defined as ‘forced into sex’) among men in the Netherlands (sexual preference was not specified), the lifetime prevalence was 6%.^24^ When including all sexual transgressive behaviour, which also includes kissing and sexual contiguity, they reported a 19% lifetime prevalence. Findings published by Haas showed a 7.7% lifetime prevalence of sexual violence among men.^20^ This percentage dropped to 1.2% when only focusing on the previous year. In our study, we find much higher percentages; in the chemsex group 12.5% of all participants reported NCS in the past year only (vs 6.0% among non-chemsex engagers; p=0.002). The difference between both groups suggests that MSM engaging in chemsex experience NCS more frequently and therefore more recently. However, the definition or concept used to describe NCS in the literature often varies and can lead to differences in findings. For example, Ward *et al* found a 42.9% prevalence among chemsex users after changing the terminology from ‘forced into sex’ to ‘non-consensual sex’ compared with 16.7% before.^16^ We therefore purposely chose to use the term ‘non-consensual sex’ to include as many forms of sexually transgressive behaviour as possible. To prevent a misinterpretation, yet also gather more in-depth information on participants’ own specific experiences, we offered some examples of NCS at the start, and enquired their own specific experiences at the end of the survey.

Among men who practise chemsex, sexual consent can be experienced as complicated and vague.^14^ In a qualitative study of Bourne *et al* among MSM engaging in chemsex, 10% of the participants reported to have overdosed on GHB with loss of consciousness and therefore could not have given consent to the sex which occurred but still did not refer to it as sexual assault or rape.^14^ In our study also, 8.8% of all chemsex engagers reported loss of consciousness due to chems, other substances and/or alcohol. Besides the feelings of guilt and shame that could go along with NCS, the cognitive dissonance theory could explain that men who attend chemsex parties might accept this ambiguity, change their attitudes towards consent (to avoid dissonance) and set a norm in which participation in such a party equals consent.^25^ This also might explain why most participants in our study who had an NCS experience reported little to no emotional distress afterwards. However, 1.8% (1 of 58) of MSM engaging in chemsex and 5.0% (5 of 103) of the MSM not engaging in chemsex rated their suffering with the highest option possible. This is in line with our finding that in the non-chemsex group, ‘being touched against one’s will’ was reported more often compared with the chemsex group. However, we do not know whether men engaging in chemsex talked beforehand about the possibility of passing out during a chemsex session and which rules would apply at this point regarding consent.

In our study, we found that men having sex with both men and women compared with men having sex with men only have higher odds of an NCS experience in the past 5 years. Earlier studies also found that bisexual individuals are more vulnerable to mental health issues, show poorer social and psychological well-being, and report a poorer overall health compared with gay men or other sexual minority.^26 27^ However, the terminology bisexuality often is used as identity measure and in our study we only asked the gender of the sex partners and therefore cannot clearly interpret this.

A strength of our study was that the survey was anonymous, therefore participants might have felt particularly open to disclose both chemsex engagement and NCS. Healthcare workers need to be aware that criminalisation, shame and stigma form barriers to disclose chemsex and NCS in institutional settings.^22 28^ It is necessary to develop strategies in which chemsex and NCS can be addressed freely. Interventions such as post-exposure prophylaxis after NCS experiences, offering pre-exposure prophylaxis to MSM engaging in sex with an increased risk of HIV transmission, or the manual ‘the Chemsex First Aid’ (a booklet that covers some general first aid practices for some specific chemsex-related emergencies) can offer support and facilitate the appropriate referrals for professional help.^23 29^ We recommend healthcare workers to be alert that NCS is an issue among MSM (regardless of chemsex engagement) and to create an open space for men to talk about NCS and offer them a referral to a professional. The Amsterdam Public Health Service has developed a chemsex support walk-in held by peers. Those peers are trained by employees of a sexual assault centre in how to handle NCS experiences from clients and refer to a sexual assault centre if wanted. Referral is also part of standard care, irrespective of chemsex engagement. These efforts help to destigmatise chemsex and communicate a welcoming attitude towards sexual assault victims in need of care.

Our study has also some limitations. To our regret, we did not ask for the quantity of NCS experiences, but solely focused on one’s last experience. Consequently, the quantity could affect the timeframe and therefore we have an incomplete picture of the impact. To study the association of chemsex engagement and NCS experiences, we used different time periods (past 6 months vs past 5 years).

In our study, we only considered crystal methamphetamine, mephedrone, and/or GHB/GBL as chemsex drugs. However, future research should take into account that within the ever-changing landscape with regional differences in availability and new drugs, also other drugs are associated with an increased risk of STIs.^5^ Another limitation of the present study is that we did not ask about polysubstance use (the use of more than one drug or type of drug consumed at the same time or sequentially) as it is another phenomenon associated with risk behaviour and psychosocial problems.^30^ Moreover, according to the syndemic theory, drug use is one of a variety of factors driving risk behaviour among MSM.^31^ Other factors such as psychosocial problems, discrimination and minority stress were not considered. It is of great importance to include the complete spectrum of risk behaviour drivers to better understand motivations for chemsex and find solutions to tackle problematic chemsex use. To also better understand the impact NCS experiences might have on one’s well-being, future research with a bigger sample size should study the associations of suffering and types of NCS. Qualitative research could also help in gaining more in-depth insights on the matter as well as on the discussion about what actually constitutes ‘consent to sex’ in a chemsex environment. These insights might lead to knowledge on how to support men confronted with NCS and prevent it from happening again.

To conclude, we showed that NCS frequently occurs among MSM, and NCS and chemsex are associated.

Whereas chemsex engagement is a new and emerging phenomenon, MSM have been victims of NCS for so long. The close associations between the two issues urge a more coherent response that need to be addressed in drug use and sexual healthcare settings. Therefore, healthcare professionals need to be open during consultation hours involving MSM in addressing chemsex, NCS (regardless of chemsex engagement), and also intimate partner violence as it is strongly associated with sexualised drug use.^32^ Referral to other healthcare facilities specialised in chemsex or NCS remains important.

**Key messages**

- A substantial proportion of men who have sex with men (MSM) (regardless of chemsex engagement) reported non-consensual sex (NCS) in the past 5 years.
- In multivariate logistic regression analysis, chemsex engagement is associated with an NCS experience.
- Among participants who reported NCS, suffering related to NCS did not differ between chemsex engagers and non-engagers.
- Sexual healthcare professionals need to address chemsex and NCS during consultations involving MSM and refer men for specialised help if deemed necessary.

10.1136/sextrans-2020-054840.supp2 Abstract translation This web only file has been produced by the BMJ Publishing Group from an electronic file supplied by the author(s) and has not been edited for content.

## Data Availability

Not applicable
