# Supplementary material for: Sexual consent and chemsex: a quantitative study on sexualised drug use and non-consensual sex among men who have sex with men in Amsterdam, the Netherlands
Source: Sex Transm Infect. 2021 Apr 8;97(4):268–75. doi: 10.1136/sextrans-2020-054840 (PMC8165144; doi:10.1136/sextrans-2020-054840)
Supplement: Supplementary data [file sextrans-2020-054840supp001.pdf]

Supplement Table 1. Specific types of non-consensual sex (NCS) experiences in relation to any amount of reported current suffering.

	**Any amount of suffering,**	**No suffering at all,**	**Total** (missing n=4)
	96 (61.1%)	61 (38.9%)	157
**Types of NCS experienced (multiple answers possible)**[1]			
Filmed/photographed without consent	13 (44.8)	16 (55.2)	29
Blackmailed (e.g. with images)	8 (100)	0	8
Used drugs against my will	16 (84.2)	3 (15.8)	19
Touched against my will	35 (64.8)	19 (35.2)	54
Had sexual contact against my will	36 (67.9)	17 (32.1)	53
Had sex without a condom against my will	30 (78.9)	8 (21.1)	38
I passed out and don't remember (under influence of chems, other substances and/or alcohol)	22 (66.7)	11 (33.3)	33
I crossed the limits of my sex partner	8 (66.7)	4 (33.3)	12
Other	3 (60.0)	2 (40.0)	5

[1] Multiple answers possible. A total of 251 types of NCS were reported by 157 individuals.

Supplement Table 2. Timeframe of one's last of non-consensual sex experiences in relation to any amount of reported current suffering.

	**Any amount of suffering, n=95 (61.1%), n (%)**	**No suffering at all, n= 61 (38.9%)**	**Total n=156 (%), n (%)** (missing n=5)
Timeframe			
Past month	10 (76.9)	3 (23.1)	13
1-3 months ago	15 (78.9)	4 (21.1)	19
3-6 months ago	10 (55.6)	8 (44.4)	18
6-12 months ago	10 (50.0)	10 (50.0)	20
>1 year ago	50 (58.1)	36 (41.9)	86
