# Supplementary material for: Sexual consent and chemsex: a quantitative study on sexualised drug use and non-consensual sex among men who have sex with men in Amsterdam, the Netherlands
Source: Sex Transm Infect. 2021 Apr 8;97(4):268–75. doi: 10.1136/sextrans-2020-054840 (PMC8165144; doi:10.1136/sextrans-2020-054840)
Supplement: Abstract translation [file sextrans-2020-054840supp002.pdf]

Sexual consent & Chemsex: A quantitative study on sexualised drug use and non-consensual sex among men who have sex with men in Amsterdam, the Netherlands

Susanne Drückler*[1] & Jilke Speulman*[1] , Martijn S. van Rooijen[1], Henry J.C. de Vries[1 2]

[1]Infectious Diseases, Public Health Service of Amsterdam, Amsterdam, North Holland, The Netherlands

[2]Department of Dermatology, Amsterdam Institute for Infection & Immunity (AI&II), Amsterdam University Medical Centers, location Academic Medical Centre, University of Amsterdam, Amsterdam, the Netherlands

Achtergrond

Chemsex (drugsgebruik tijdens de seks) is de afgelopen jaren toegenomen onder mannen die seks hebben met mannen (MSM). Daarnaast lijkt seksueel grensoverschrijdend gedrag (SGG) vaak voor te komen onder invloed van chemsex. In deze studie onderzochten wij het verband tussen SGG in chemsex.

Methoden

We vroegen gebruikers van verschillende gay datingplatforms een vragenlijst in te vullen over chemsex gebruik in de afgelopen 6 maanden (crystal methamphetamine, mephedrone en / of gamma-hydroxybutyraat / gamma-butyrolacton gebruik) en over hun ervaringen met SGG in de afgelopen 5 jaar (seks zonder wederzijds goedvinden of nare seksuele ervaringen). Associaties werden gemeten met behulp van χ^2-test, Fisher's exact-test en multivariabele logistische regressie.

Resultaten

In totaal vulden 891 MSM de vragenlijsten in, waarvan 30,6% (273 / 891) chemsex gebruikte in de afgelopen 6 maanden. MSM die aan chemsex deden meldde in 21,2% SGG ervaringen tegenover 16,7% van de mannen die niet aan chemsex deden (p=0,109).

MSM die SGG rapporteerden en aan chemsex deden meldde minder vaak dat ze tegen hun wil waren aangeraakt, in vergelijking met MSM die niet aan chemsex deden (22,4% versus 39,8%; pp = 0,036). Daarentegen meldden MSM die aan chemsex deden vaker dat ze flauw vielen en zich niet meer konden herinneren wat er gebeurde tijdens chemsex (resp. 41,4% versus 8,7%; pp <0,001). De lijdenslast na een SGG ervaring verschilde niet tussen MSM die aan chemsex deden en MSM die niet aan chemsex deden (p = 0,539); en in 77,1% werd lijdenslast als helemaal afwezig of weinig ervaren. In de multivariabele regressieanalyses was chemsex gebruik in de afgelopen zes maanden geassocieerd met SGG (aOR, 1,46; 95% CI 1,01 tot 2,11).

Conclusie

Een aanzienlijk deel van de MSM (ongeacht chemsex gebruik) rapporteerde SGG in de afgelopen 5 jaar. In multivariate logistische regressieanalyse werd chemsex gedrag geassocieerd met seksueel grensoverschrijdende ervaringen. Lijdenslast na een seksueel grensoverschrijdende ervaring verschilde niet tussen MSM die wel of geen chemsex gebruikten.

Professionals in de seksuele gezondheidszorg moeten chemsex en SGG bespreekbaar maken tijdens consulten met MSM en deze mannen indien nodig doorverwijzen naar verslaving- of mentale gezondheidshulpverlening.
